# Lipid accumulation product and visceral adiposity index for incidence of cardiovascular diseases and mortality; results from 13 years follow‐up in Isfahan cohort study

**DOI:** 10.1002/osp4.713

**Published:** 2023-10-02

**Authors:** Mohammad Fakhrolmobasheri, Amir Parsa Abhari, Maryam Heidarpour, Saina Paymannejad, Mahsa Pourmahdi‐Boroujeni, Amir Sepehr Saffari, Paria Okhovat, Hamidreza Roohafza, Masoumeh Sadeghi, Najmeh Rabanipour, Davood Shafie, Nizal Sarrafzadehgan

**Affiliations:** ^1^ Heart Failure Research Center Cardiovascular Research Institute Isfahan University of Medical Sciences Isfahan Iran; ^2^ Isfahan Endocrine and Metabolism Research Center Isfahan University of Medical Sciences Isfahan Iran; ^3^ Student Research Committee Isfahan University of Medical Sciences Isfahan Iran; ^4^ Cardiac Rehabilitation Research Center Cardiovascular Research Institute Isfahan University of Medical Sciences Isfahan Iran; ^5^ Department of Biostatistics and Epidemiology School of Health Isfahan University of Medical Sciences Isfahan Iran; ^6^ Isfahan Cardiovascular Research Center, Cardiovascular Research Institute, Isfahan University of Medical Sciences Isfahan Iran

**Keywords:** abdominal obesity, anthropometry, cardiovascular diseases, mortality

## Abstract

**Background:**

/Aims: Visceral adiposity index (VAI) and lipid accumulation product (LAP) are novel anthropometric indices that have shown an association with metabolic syndrome; however, limited data are available regarding the predictive performance of these indices for the incidence of cardiovascular diseases (CVD) and mortality.

**Methods:**

This study was performed on the data retrieved from Isfahan Cohort Study (ICS). ICS is an ongoing population‐based cohort study conducted in 3 counties in central Iran. Pearson correlation analysis was performed between LAP, VAI, and metabolic parameters. Cox regression analysis and receiver operative characteristics (ROC) curve analysis were performed in order to evaluate the ability of VAI and LAP for the incidence of CVD, CVD‐associated mortality, and all‐cause mortality. We further compared the predictive performance of VAI and LAP with body mass index (BMI).

**Results:**

LAP and VAI were significantly correlated with all metabolic variables, including blood pressure, fasting blood glucose, and lipid profile components. Univariate regression analysis indicated a significant association between LAP and VAI and CVD incidence. In multivariate analysis, only VAI was significantly associated with CVD incidence. Regarding CVD mortality, only VAI in the multivariate analysis revealed a significant association. Interestingly, Both VAI and LAP were negatively associated with all‐cause mortality. ROC curve analysis indicated the superior performance of LAP and VAI for predicting CVD incidence compared to BMI; however, BMI was better in predicting all‐cause mortality.

**Conclusion:**

Compared to BMI, LAP and VAI have better predictive performance for the incidence of CVD. In contrast, BMI was superior to VAI and LAP in the prediction of all‐cause mortality.

## Graphical abstract

Compared to BMI, Lipid Accumulation Product and Visceral Adiposity Index are better predictors of the incidence of cardiovascular diseases in a population from central Iran.

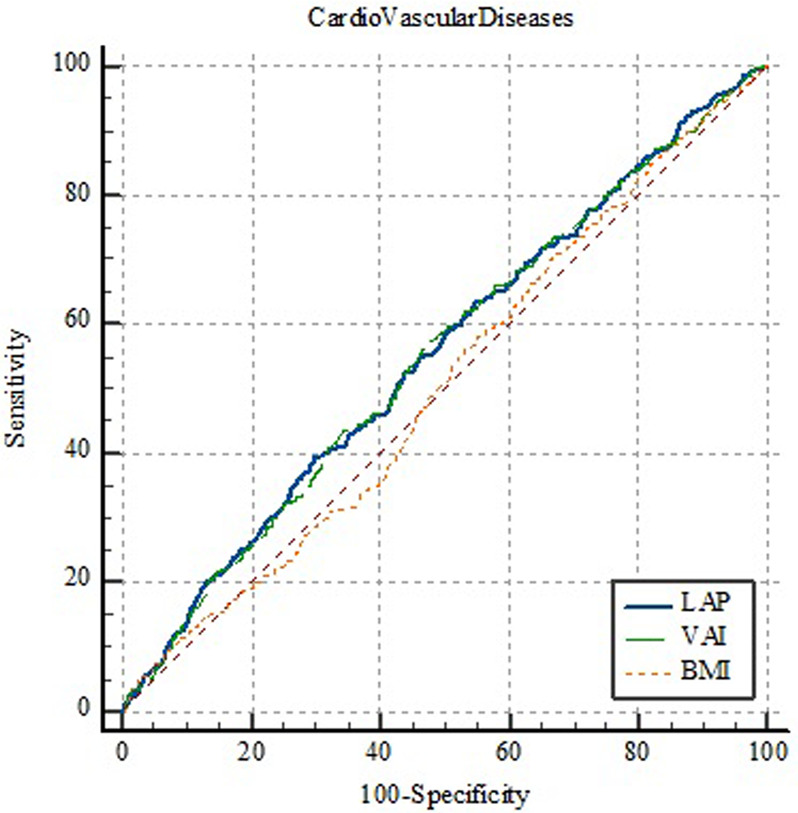

## 1 INTRODUCTION

Cardiovascular diseases (CVDs) are the leading cause of mortality and morbidity worldwide. It is estimated that 23.6 million people will die of CVD annually until 2030.^1^ Obesity is one of the main risk factors associated with CVDs. Recently, the classic definition of obesity, which is defined as a body mass index (BMI) over 30, has become a subject of debate.^2^ It has been established that dysfunctional visceral adipose tissue plays a superior role compared to the subcutaneous adipose tissue in the pathogenesis of obesity‐related diseases as it promotes a more robust systemic inflammation by producing pro‐inflammatory cytokines and a lower amount of leptin.^3^ The lack of BMI accuracy in the differentiation of the lean mass from the fat mass as well as the subcutaneous fat from the visceral fat accentuated the importance of proposing novel methods for estimating dysfunctional adipose tissue accumulation.^4^

Radiological assessment of body fat distribution using magnetic resonance imaging or computed tomography scan could precisely indicate central and visceral obesity; however, these methods are of high cost and require great resources.^5^ As a result, novel anthropo‐metabolic indices have been developed as easily available and low‐cost indicators of central and visceral obesity.^6^ Lipid accumulation product (LAP) is a Visceral adiposity index (VAI) that is calculated using waist circumference (WC) and triglycerides (TG). A number of studies have demonstrated the predictive performance of LAP for the incidence of CVDs.^7^, ^8^ Moreover, LAP could be used as an indicator of diabetes mellitus (DM) and metabolic syndrome (MetS).^9^, ^10^ The VAI is a sex‐specific index calculated using the common parameters of lipid profile [TG and high‐density lipoprotein cholesterol (HDL‐C)] and simple anthropometric measurements (BMI and WC).^11^, ^12^ VAI indicates fat distribution and visceral adipose tissue dysfunction.^12^ Accordingly, VAI has indicated a great predictive performance for the incidence of CVDs.^11^ Although numerous studies have evaluated VAI and LAP's ability to predict MetS, DM, and CVDs, few studies have compared these two indexes in predicting the incidence of CVDs and CVD‐related mortality.^13^, ^14^, ^15^, ^16^ In this study, we compared the predictive performance of VAI and LAP for the incidence of CVDs, all‐cause, and CVD‐related mortality in a population‐based cohort study in central Iran. We further attempted to compare the prognostic performance of VAI and LAP with BMI as the classic and widely used index of obesity.

## 2 METHODS

### 2.1 Study design and participants

Isfahan cohort study (ICS) was a population‐based, longitudinal study performed by the Isfahan Cardiovascular Research Institute, a World Health Organization collaborating center, in Isfahan, Arak, and Najafabad in central Iran. The study population was selected using multi‐stage cluster random sampling from all healthy, non‐pregnant individuals ≥35 years old. The study population was selected in order to reflect the age, sex, and rural/urban distribution of the target population. Detailed methods and designs of the ICS are published separately.^17^ This study was performed on 13‐year follow‐up results of ICS. The institutional review board of Isfahan University of Medical Sciences reviewed the study protocol and an ethical approval code was obtained (IR.ARI.MUI.REC.1401.161) for the research.

### 2.2 Outcomes

The primary outcomes of the study were the incidence of CVDs, all‐cause mortality, and cause‐specific mortality, while secondary outcomes were risk factors of CVDs, including the incidence of hypertension (HTN), DM, and MetS.

### 2.3 Baseline survey

At baseline, after obtaining the written informed consent, all participating individuals had a face‐to‐face interview with trained staff nurses answering a pre‐defined questionnaire. The questionnaire included questions obtaining data surrounding demographics, socioeconomic status, quality of life, personal medical history, daily physical activity, psychological distress, sleeping pattern, dietary habits, and smoking status. After that, a comprehensive physical examination by trained physicians was performed on the participants. Vital signs, cardiopulmonary system evaluations, and anthropometric measurements were examined based on a pre‐defined check‐list designed for ICS. 12 h fasting blood samples were used to measure fasting blood glucose (FBS), lipid profile, C‐reactive protein and complete blood count (CBC). Also, 2 h of postprandial serum glucose was measured for all participants.

### 2.4 Follow‐up surveys

Every 2 years, telephone follow‐up surveys were performed to record the incidence of primary outcomes. Phone calls were performed by trained staff using a pre‐specified check list to obtain information regarding the time and place of the incidence of the primary outcomes. Furthermore, a comprehensive physical examination, laboratory evaluation, and verbal interview were performed every 5 years to evaluate the incidence of secondary outcomes.

### 2.5 Outcome ascertainment

In the case of hospitalization, information surrounding the time, place, and final diagnosis of the hospitalization episode was recorded during each 2‐year telephone interview. Information related to the cause and place of death was recorded regarding the death of a participant. If the participant was hospitalized before death, information about the hospitalization period and final diagnosis before death was obtained from the family survivors. Death certificates were also attained from the provincial mortality database. Moreover, staff nurses collected documents about hospitalization events. Finally, an outcome adjudication panel with four cardiologists and two neurologists evaluated the available documents regarding the incidence of death or hospitalization.

### 2.6 Measurements

We used a validated Persian version of the food frequency questionnaire for assessing dietary habits.^18^ Further, we calculated each participant's global dietary index (GDI) score using the GDI scoring system.^19^ Moreover, a validated Persian format of the international physical activity questionnaire was used to assess each participant's daily physical activity.^20^

Anthropometric measurements were performed during the physical examination while the study participants wore light clothes without shoes. We used a standard calibrated scale and tape with similar brands. This study defined WC as the “smallest circumference at the costal margin or below.” Blood pressure (BP) measurement was performed twice in each episode of physical examination at a 15‐min interval using calibrated mercury sphygmomanometers of the same brand. The mean systolic BP (SBP) and diastolic BP (DBP) of the two measurements were recorded as the patients' SBP and DBP.

After obtaining 10 mL of fasting blood, the samples were immediately transported to the central laboratory of Isfahan Cardiovascular Research Institute, Isfahan, Iran. Two auto‐analyzers were used for the CBC: Eppendorf, Hamburg, Germany, in 2001 and Hitachi 902, Japan, in 2007. FBS, HDL‐C, and TG levels were measured using special enzyme assay kits (Immunodiagnostic, Frankfurt, Germany).

### 2.7 Definitions

Abnormal components of lipid profile were defined as total cholesterol (TC) ≥200 (mg/dl), low‐density lipoprotein‐cholesterol (LDL‐C) ≥160 mg/dl, HDL‐C <40 mg/dl in men and <50 mg/dl in women, and TG ≥150 mg/dl.^21^

VAI was calculated separately for males and females as:

Male: $\left(\frac{WC}{39.68}+BMI\times 1.88\right)\times \left(\frac{total\;TG}{1.03}\right)\times \frac{1.31}{HDL}$
Female: $\left(\frac{WC}{36.58}+BMI\times 1.89\right)\times \left(\frac{total\;TG}{0.81}\right)\times \frac{1.52}{HDL}$

LAP was calculated separately for males and females as:

Male: (WC − 65) × TG
Female: (WC − 58) × TG

Cardiovascular events were defined as myocardial infarction (MI), fatal and non‐fatal stroke, unstable angina (UA), and sudden cardiac death (SCD) in accordance with modified criteria of the WHO expert committee.^22^ Ischemic heart diseases (IHD) include MI, UA, and SCD. The definition of acute MI was defined as; the presence of at least two of the following criteria:

1) Typical chest pain lasting more than 30 min,
2) ST elevation  >0.1 mV in at least 2 adjacent electrocardiograph leads,
3) Rise in serum levels of cardiac biomarkers [including creatine kinase (CK), CK‐myoglobin binding (CK‐MB), CK‐MB mass (CK‐MBm), or troponin (cTn)]. The diagnostic values of these markers are as follows: cTn > CK‐MBm > CK‐MB > CK.

UA was defined as new onset typical chest discomfort lasting longer than 20 min within the 24 h preceding hospitalization or a change in the usual pattern of angina. Diagnosis of UA was based on dynamic ST‐segment or T‐wave changes in ≥2 ECG leads.^23^ SCD was defined as Death within 1 h of onset, a witnessed cardiac arrest, or abrupt collapse not preceded by >1 h of symptoms. According to the WHO definition, stroke is a rapidly developing focal or global neurological dysfunction lasting longer than 24 h with a probable vascular origin.^24^

### 2.8 Statistical analysis

Baseline characteristics of the participants, including demographic features and laboratory measures, are presented as mean ± standard deviation (SD) for continuous variables and frequency (percentage) for categorical variables. These variables are shown according to VAI and LAP cut‐off points, and intergroup comparisons were performed using *T*‐test (Mann‐Whitey *U* test in case of non‐normal distribution) and Chi‐square test, where appropriate. Partial correlation analysis assessed the correlation between VAI and LAP and metabolic markers, including SBP, DBP, FBS, HDL‐C, and LDL‐C. The receiver operating characteristics (ROC) curve and Youden's index were used to identify the optimal cut‐off values for VAI and LAP. In order to compare the prognostic performance of VAI, LAP, and BMI, pairwise comparison of ROC curves was carried out for CVD incidence, CVD mortality, and all‐cause mortality. The area under the curve (AUC) was used to measure how well these indices could prognosticate our outcomes, with an AUC of 1.0 showing perfect predictability and an AUC of 0.5 implying that the discriminatory accuracy is not better than the chance. Multivariate and univariate Cox regression analysis was conducted to obtain crude and adjusted hazard ratio for the outcomes. Statistical analyses were implemented in the SPSS software (version 26, IBM), and MedCalc® Statistical Software version 20.104 (MedCalc Software Ltd, Ostend, Belgium; https://www.medcalc.org; 2022) was used to perform ROC curve analysis and obtain Youden's index, sensitivity, and specificity of the anthropometric indices.

## 3 RESULTS

### 3.1 Demographic, clinical, and laboratory data of the study population

A total of 4353 (1995 males and 2358 females, female o to male ratio of 1.2:1) subjects participated in this study. The mean age of the participants was 51.78 ± 12.17 years. The differences in age, education, CVD mortality, and all‐cause mortality between participants with high values of VAI and LAP and those with low values of these indices were not significant; the difference in HDL‐C between high and low LAP was not meaningful either. However, the differences in sex distribution, GDI, daily physical activity, LDL‐C, TC, TG, FBS, systolic and diastolic HTN, smoking, family history of premature CVD (FH‐CVD), and CVD incidence between high and low VAI and LAP were significant. These findings are thoroughly presented in Table 1.

**TABLE 1:** Compare baseline characteristics of the study population according to VAI and LAP.

Variables	Total (*n* = 4353)	VAI			LAP		
		<2.83 (*n* = 2268)	>2.83 (*n* = 2085)	*p*‐value*	<96.64 (*n* = 3066)	>96.64 (*n* = 1292)	*p*‐value*
Age	51.78 ± 12.17	51.65 ± 12.55	51.88 ± 11.71	0.52	51.55 ± 12.49	52.26 ± 11.34	0.07
Sex							
Male	1995 (45.8)	1286 (56.7)	709 (34)	**<0.0001**	1564 (51)	433 (33.5)	**<0.0001**
Female	2358 (54.2)	982 (43.3)	1376 (66)		1502 (49)	859 (66.5)	
Education (year)							
0–5	3183 (73.3)	1636 (72.3)	1547 (74.3)	0.34	2225 (72.7)	961 (74.5)	0.21
6–12	910 (20.9)	492 (21.8)	418 (20.1)		646 (21.1)	266 (20.6)	
>12	252 (5.8)	134 (5.9)	118 (5.7)		189 (6.2)	63 (4.9)	
Global dietary index	1.01 ± 0.25	1.03 ± 0.24	0.99 ± 0.26	**<0.0001**	1.03 ± 0.24	0.97 ± 0.27	**<0.0001**
Physical activity (daily)	834.20 ± 537.27	891.13 ± 552.94	774.31 ± 513.31	**<0.0001**	871.87 ± 547.01	747.50 ± 503.08	**<0.0001**
HDL	46.94 ± 10.51	49.94 ± 10.37	43.69 ± 9.69	**<0.0001**	47.08 ± 10.37	46.62 ± 10.88	0.19
LDL	129.18 ± 43.08	123.23 ± 40.40	135.69 ± 44.98	**<0.0001**	125.26 ± 40.76	138.59 ± 46.87	**<0.0001**
TC	214.52 ± 52.34	198.84 ± 45.45	231.63 ± 54.07	**<0.0001**	201.61 ± 45.28	245.24 ± 55.21	**<0.0001**
Triglyceride	192.13 ± 103.18	128.62 ± 43.89	261.22 ± 104.90	**<0.0001**	146.55 ± 56.23	300.18 ± 108.74	**<0.0001**
FBS	89.99 ± 33.82	85.80 ± 28.95	94.51 ± 37.80	**<0.0001**	85.78 ± 28.55	100.02 ± 42.30	**<0.0001**
Hypertension systolic	122.61 ± 21.56	120.08 ± 21	125.40 ± 21.83	**<0.0001**	120.07 ± 20.79	128.69 ± 22.18	**<0.0001**
Hypertension diastolic	78.82 ± 11.77	77.44 ± 11.37	80.35 ± 12.03	**<0.0001**	77.50 ± 11.26	81.98 ± 12.39	**<0.0001**
Smoke							
Yes	701 (16.1)	424 (18.7)	277 (13.3)	**<0.0001**	545 (17.8)	157 (12.2)	**<0.0001**
No	3647 (83.9)	1842 (81.3)	1805 (86.7)		2518 (82.2)	1133 (87.8)	
FH‐CVD							
Yes	1177 (27)	547 (24.1)	630 (30.2)	**<0.0001**	763 (24.9)	417 (32.3)	**<0.0001**
No	3176 (73)	1721 (75.9)	1455 (69.8)		2303 (75.1)	875 (67.7)	
CVD follow‐up duration (month)	112.57 ± 51.99 (1–165)	111.14 ± 51.88	114.36 ± 51.96	0.07	110.51 ± 51.82	117.55 ± 51.90	**<0.0001**
CVD/All‐cause mortality follow‐up duration (month)	114.47 ± 49.10 (1–160)	112.14 ± 49.63	117.22 ± 48.25	**0.002**	111.75 ± 49.49	120.89 ± 47.41	**<0.0001**
Incident CVD							
Yes	408 (11.8)	176 (9.8)	232 (13.9)	**<0.0001**	248 (10.4)	162 (15.1)	**<0.0001**
No	3057 (88.2)	1615 (90.2)	1442 (86.1)		2147 (89.6)	913 (84.9)	
CVD mortality							
Yes	152 (4.4)	74 (4.1)	78 (4.7)	0.45	104 (4.3)	49 (4.6)	0.77
No	3313 (95.6)	1717 (95.9)	1596 (95.3)		2291 (95.7)	1026 (95.4)	
All‐cause mortality							
Yes	407 (11.7)	221 (12.3)	186 (11.1)	0.26	297 (12.4)	112 (10.4)	0.09
No	3058 (88.3)	1570 (87.7)	1488 (88.9)		2098 (87.6)	963 (89.6)	

*Note*: Data are represented as mean ± SD or frequency (percent). Bold values mean that they are statistically significant (*p*‐value less than 0.05).

Abbreviations: FBS, fasting blood sugar; FH‐CVD, family history of cardiovascular disease; HDL, high‐density lipoprotein; LAP, Lipid Accumulation Product; LDL, low‐density lipoprotein; TC, total cholesterol; VAI, Visceral Adiposity Index.

*p values were derived from independent *t*‐test and chi‐square test.

### 3.2 Partial correlation between each anthropometric index and metabolic parameters

After adjusting for age and smoking status, LAP and VAI were significantly correlated with all metabolic variables, including SBP, DBP, FBS, HDL‐C, and LDL‐C. FBS and SBP had the highest correlation coefficients, and HDL‐C was negatively correlated with LAP. These findings are comprehensively shown in Table 2.

**TABLE 2:** Partial correlation between VAI, LAP, and metabolic parameters.

	SBP		DBP		FBS		HDL		LDL		VAI	
	Coe	Sig	Coe	Sig	Coe	Sig	Coe	Sig	Coe	Sig	Coe	Sig
LAP	0.214	0.0001	0.197	0.0001	0.227	0.0001	−0.031	0.043	0.178	0.0001	0.836	0.0001
VAI	0.147	0.0001	0.136	0.0001	0.194	0.0001	‐	‐	0.157	0.0001	1	‐

Abbreviations: COE, Correlation Coefficient; DBP, Diastolic Blood Pressure; FBS, Fasting Blood Sugar; HDL, High‐Density Lipoprotein; LDL, Low‐Density Lipoprotein; SBP, Systolic Blood Pressure.

### 3.3 Risk of CVD incidence and mortality

Univariate Cox regression analyses showed a significant association between LAP and VAI and CVD incidence (HR: 1.36 [1.11–1.66], *p*‐value of 0.002; HR: 1.37 [1.12–1.66], *p*‐value of 0.002, respectively). Although after adjusting for age, sex, physical activity, FBS, smoking, and systolic HTN, VAI remained significantly associated with CVD incidence (HR: 1.32 [1.07–1.62, *p*‐value of 0.009]), cox regression revealed an insignificant association of LAP with CVD incidence (HR: 1.13 [0.91–1.39, *p*‐value of 0.26]). Interestingly, there was a reverse association between all‐cause mortality and LAP/VAI in univariate and multivariate. However, these two indices were not significantly associated with CVD mortality, except for VAI in multivariate analysis (HR: 1.47 [0.99–2.19, *p*‐value of 0.04]). Details of the analyses, along with the adjusted covariates, are presented in Table 3.

**TABLE 3:** Hazard ratios of incident CVD, CVD mortality, and all‐cause mortality according to VAI and LAP.

		CVD incident		CVD mortality		All‐cause mortality	
Variable		Univariate HR (95% CI)	Multivariate HR (95% CI)	Univariate HR (95% CI)	Multivariate HR (95% CI)	Univariate HR (95% CI)	Multivariate HR (95% CI)
VAI	2.83>	1.37 (1.12–1.66)	1.32 (1.07–1.62)^a^	1.17 (0.79–1.71)	1.47 (0.99–2.19)^c^	0.77 (0.63–0.94)	0.74 (0.58–0.93)^f^
	*p*	0.002	0.009	0.42	0.04	0.01	0.01
	−2 log likelihood	6338.55	5982.73	2376.68	2142.43	6363.39	5909.61
LAP	>96.64	1.36 (1.11–1.66)	1.13 (0.91–1.39)^b^	1.061 (0.76–1.46)	1.13 (0.80–1.59)^d^	0.62 (0.51–0.77)	0.65 (0.51–0.81)^e^
	*p*	0.002	0.26	0.72	0.47	0.0001	0.0001
	−2 log likelihood	6369.91	6011.33	2392.64	2157.34	6384.26	5933.72

^a^ Adjusted for age, sex, physical activity, FBS, systolic hypertension and smoking.

^b^ Adjusted for age, sex, physical activity, HDL, FBS, systolic hypertension and smoking.

^c^ Adjusted for age, sex, global dietary index, physical activity, and FBS.

^d^ Adjusted for age, sex, global dietary index, physical activity, and FBS.

^e^ Adjusted for age, sex, global dietary index, physical activity, diastolic hypertension, and FBS.

^f^ Adjusted for age, sex, HDL, global dietary index, physical activity, diastolic hypertension, and FBS.

### 3.4 Comparing the predictive performance of VAI, LAP, and BMI according to ROC curve analysis

**FIGURE 1:**
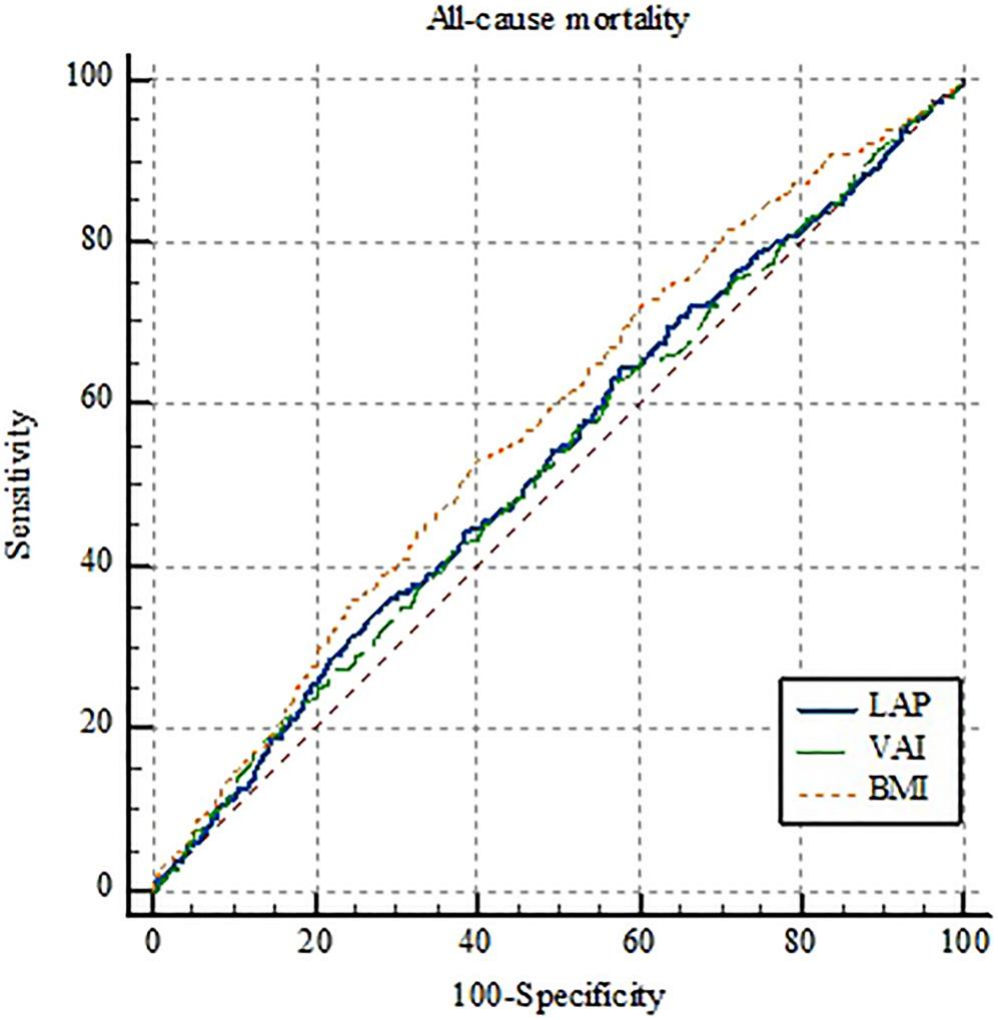
Receiver operating characteristics (ROC) curve for lipid accumulation product (LAP), visceral adiposity index (VAI), and body mass index (BMI) according to all‐cause mortality.

**FIGURE 2:**
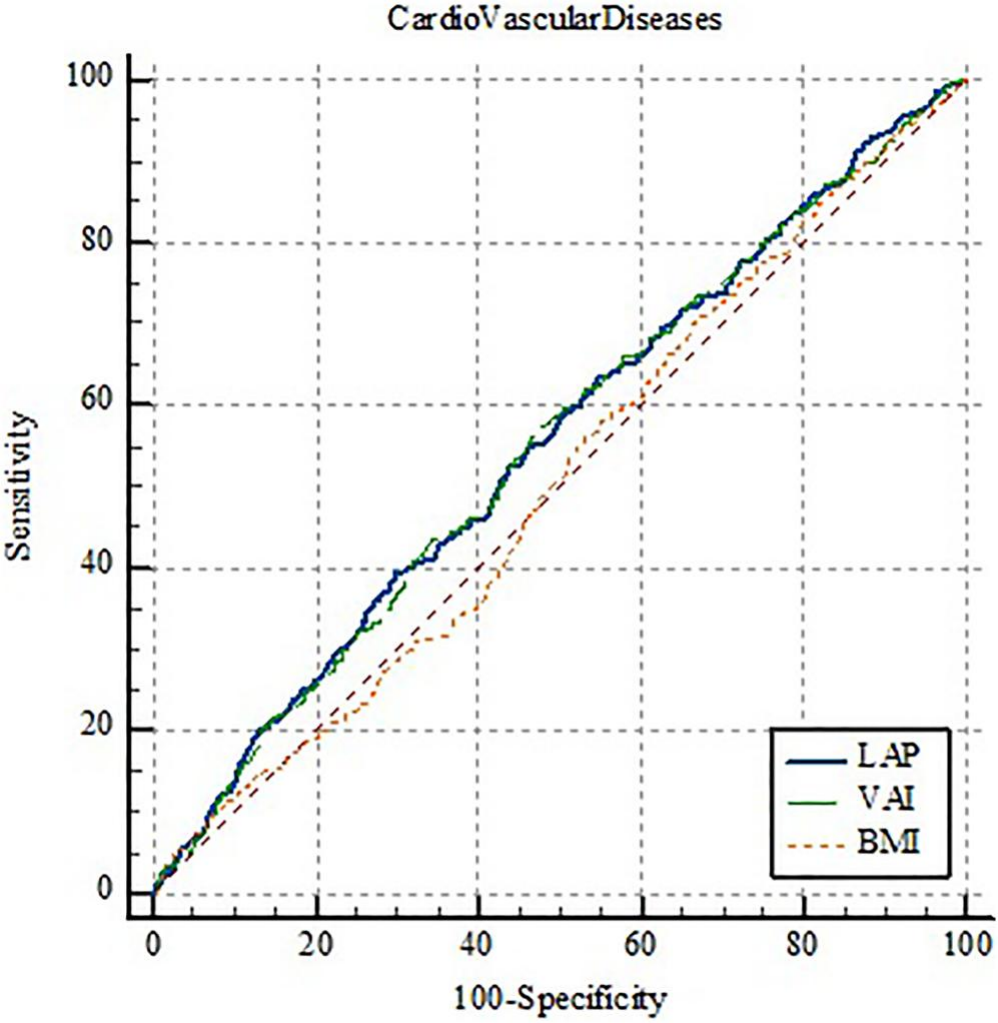
Receiver operating characteristics (ROC) curve for lipid accumulation product (LAP), visceral adiposity index (VAI), and body mass index (BMI) according to the incidence of cardiovascular diseases.

**FIGURE 3:**
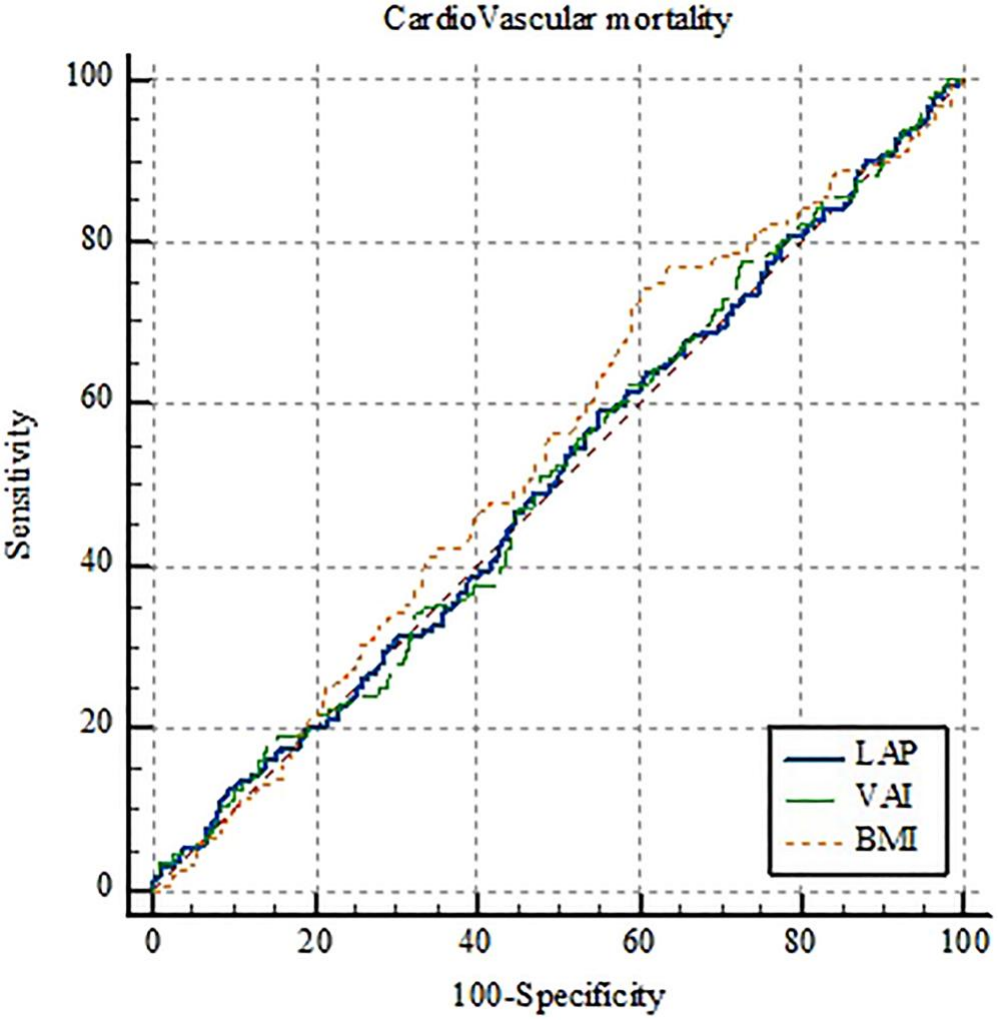
Receiver operating characteristics (ROC) curve for lipid accumulation product (LAP), visceral adiposity index (VAI), and body mass index (BMI) according to cardiovascular mortality.

The AUC of LAP and VAI as well as the optimal cut‐off points, sensitivity, specificity, and Youden's indices are shown in Table 4. Moreover, the details of ROC curve analysis for BMI, LAP, and VAI are represented in Table 5. Since all AUCs were between 0.5 and 0.6, BMI, LAP, and VAI failed to perform strong classification according to CVD incidence, CVD mortality, and all‐cause mortality. Regarding the incidence of CVD, VAI, and LAP have shown significantly greater predictive performance compared to BMI; however, there was no significant difference between VAI and LAP (Figure 1). Interestingly, none of the indices were able to predict CVD‐associated mortality (Figure 2). Furthermore, compared to VAI and LAP, BMI has indicated a significantly greater ability to predict all‐cause mortality, whereas neither VAI nor LAP was not able to demonstrate a statistically significant predictive performance (Figure 3). Details of the pairwise comparison of ROC curves are represented in Tables S1–S3.

**TABLE 4:** Receiver operative characteristic curves and cut off values of VAI and LAP for incident CVD, CVD mortality, and all‐cause mortality.

		AUC	Cut‐off values	Sensitivity (%)	Specificity (%)	Youden's index	*p*‐value
Incident CVD	VAI	0.55	2.83	56.86	53.06	0.1	0.0005
	LAP	0.55	96.64	39.51	70.20	0.1	0.0003
CVD mortality	VAI	0.509	1.89	77.6	27.1	0.04	0.69
	LAP	0.509	60.6	59.48	44.83	0.04	0.7
All‐cause mortality	VAI	0.527	3.1	63.1	42.6	0.057	0.07
	LAP	0.534	40.33	31.8	75.3	0.07	0.02

Abbreviation: AUC, area under the receiver operating characteristic curve.

**TABLE 5:** Results of receiver operating characteristics (ROC) curve analysis for VAI, LAP, and BMI according to CVD event, CVD mortality, and all‐cause mortality.

		LAP	VAI	BMI
CVD incidence	AUC	0/555	0/553	0/507
	SE	0/0152	0/0152	0/0149
	95% CI	0/539 to 0/572	0/536 to 0/569	0/490 to 0/524
	Sig	0/0003	0/0005	0/6436
CVD mortality	AUC	0/509	0/509	0/539
	SE	0/0238	0/0237	0/0230
	95% CI	0/492 to 0/526	0/492 to 0/526	0/522 to 0/556
	Sig	0/6999	0/6950	0/0903
All‐cause mortality	AUC	0/534	0/527	0/577
	SE	0/0154	0/0154	0/0149
	95% CI	0/517 to 0/550	0/511 to 0/544	0/560 to 0/593
	Sig	0/0291	0/0747	<0/0001

Regarding the obtained cut‐off values of VAI and LAP, the highest sensitivity refers to the predictability of VAI for CVD mortality (AUC: 0.509, cut‐off value: 1.89, and sensitivity: 77.6%), and the highest specificity refers to the predictability of LAP for all‐cause mortality (AUC: 0.534, cut‐off: 40.33, and specificity: 75.3%).

## 4 DISCUSSION

The present study investigated the predictive performance of VAI and LAP as visceral fat markers for CVDs in a large sample of Iranians. CVDs and all of the comorbidities seem to be of the same nature as these disorders are strongly associated with overweight and obesity. Visceral adipose tissue accumulation results in increased immune response and vasoconstrictor mediator secretion, whereas subcutaneous adipose tissue expansion is less dangerous. Therefore, fat distribution rather than overall body weight is a crucial determining factor of CVDs and their comorbidities.^25^

We found that a higher VAI and LAP score is associated with an increased CVD incidence rate. ROC analyses revealed that although the AUC for VAI and LAP is more than 0.5, neither VAI nor LAP could poorly predict CVD incidence. In this regard, a large‐scale American study analyzed the data extracted from the National Health and Nutrition Examination Survey from 1998 to 2018 to examine the association between VAI and CVD incidence. A non‐linear positive correlation was observed for angina, heart attack, stroke, and coronary heart disease but not for heart failure. Similarly, the results suggest a poor predictive role of VAI and LAP for CVD incidence (AUC for both indices was 0.5–0.6).^26^ The anthropo‐metabolic indices can assert effects on the risk of CVDs both directly and indirectly. The direct effect of VAI was well described in a cross‐sectional study on a large number of healthy Korean individuals, which revealed a significant association between VAI and subclinical atherosclerosis by evaluating the coronary artery calcium score. The study observed that individuals with higher VAI values had a significantly higher amount of calcification in coronary arteries and therefore had an increased risk of developing CVD.^27^

Moreover, a Brazilian study on a large number of healthy participants reported that patients with a higher intima‐media thickness of the carotid artery had higher LAP and VAI scores and therefore an increased risk for developing subclinical atherosclerosis and CVDs.^28^

Similar results were observed for the LAP index in a study performed on peri‐menopausal women above 40 years of age in Thailand^29^; however, VAI was not correlated with CVD incidence in this study. The heterogeneity of the results could be due to the exclusiveness of the participants. Therefore, older age, sex, menopause‐associated changes in body composition and lipid metabolism,^30^ and the lower amount of estrogen, which plays a protective role against the visceral accumulation of fat and CVD development,^31^ should be considered in interpreting the results.

Fiorentino et al. evaluated the direct effects of VAI, LAP, and two other anthropo‐metabolic indices on CVD incidence. Pulse pressure and intima‐media thickness of the carotid artery were measured as indicators of subclinical vascular damage. Accordingly, all four indices showed a similar ability in detecting vascular atherosclerosis defined by increased carotid artery intima‐media thickness; however, LAP had the greatest capability to recognize elevated vascular stiffness defined by pulse pressure ≥60 mm Hg. Therefore, the authors concluded that assessing the LAP index in clinical practice should be preferred over VAI for better CVD risk stratification.^32^ We did not observe any significant difference between LAP and VAI for CVD incidence in the present study. On the other hand, after adjusting for confounding variables, only VAI remained significantly associated with CVD incidence. This inconsistency could be due to the different natures of the studies, as we directly measured the clinical rate of CVDs among the population. However, the aforementioned study implied measures to examine the rate of subclinical vascular damage as the etiological factor of CVD.

The direct association between anthropo‐metabolic indices and CVD incidence could be explained by the distinct biological characteristics of visceral adipose tissue. Compared to its subcutaneous counterpart, visceral fat is associated with a lower HDL‐C/LDL‐C ratio, lower adiponectin plasma concentration, and higher lipolysis. High lipolysis produces small and dense LDL‐C particles with a great ability to infiltrate into the subendothelium. Moreover, low HDL‐C and adiponectin prompt the activation of macrophages and the formation of foam cells, which eventually produce cytokines and growth factors contributing to the development of vascular damage and therefore CVDs.^33^, ^34^

Furthermore, we discovered a positive association between VAI and LAP with CVD comorbidities that could explain the indirect effect of anthropo‐metabolic indices on developing CVDs. We observed that FBS and SBP had the highest correlation coefficients with VAI and LAP scores.

The correlation between these anthropo‐metabolic indices and impaired glycemic status has been described previously.^16^, ^35^ It is demonstrated that VAI could be used as an appropriate predictive surrogate index of insulin resistance, which is currently measured by the gold standard technique of hyperinsulinemic‐euglycemic clamp.^36^, ^37^ Moreover, VAI could be an independent predictor of pre‐diabetes and type 2 DM.^38^, ^39^ Notably, Fiorentino et al. reported that the LAP index is a more reliable discriminator of insulin resistance in clinical settings than VAI.^32^

A positive association between SBP and DBP with anthropo‐metabolic indices has also been shown. A large Taiwanese cohort study confirmed that VAI and LAP could be suitable predictors of HTN, especially in women.^14^ Nevertheless, several articles claimed there is no superiority in applying these novel anthropo‐metabolic indices over the traditional ones, including BMI and WC, for predicting HTN.^40^, ^41^

Higher VAI and LAP scores represent increased visceral fat, including the perirenal space and renal sinuses. The fat can compress the kidneys, increase intrarenal pressure, and reduce medullary blood flow, leading to the renin‐angiotensin‐aldosterone system's (RAAS) activation, sodium reabsorption, secretion, and ultimately HTN.^42^

Although not significant for CVD mortality, there was a significant association between VAI and LAP and all‐cause mortality in both univariate and multivariate analyses. Of note, according to ROC analysis, these indices failed to predict CVD and all‐cause mortality. A higher visceral‐to‐subcutaneous fat ratio is reported to be a reliable predictor of all‐cause mortality.^43^ In this regard, the predictive performance of the two indices has already been evaluated among populations with a specific underlying characteristic. The correlation of VAI with all‐cause mortality was positive among hemodialysis patients^44^ and negative among people with peripheral artery disease.^45^ It showed a J‐shaped fashion among patients with chronic kidney disease.^46^ In addition, a study on patients with a high cardiovascular risk reported LAP as an independent risk factor for all‐cause mortality, with the strongest association in patients without DM, men younger than 50, and women.^47^ A similar study on a high‐risk population referred for coronary evaluation suggested a positive association of LAP and all‐cause mortality in postmenopausal women but not in men.^48^

Having said all that, data regarding the healthy population is scarce. A large‐scale nationwide study from the UK reported that VAI was significantly correlated with an increased risk of all‐cause mortality.^49^ Although this finding is consistent with our study, a J‐shaped link correlation was observed in a sample of elderly Americans with a mean age of 73.4 years.^50^ Nevertheless, the latter could not be confidently compared with our results due to the significant difference in age and the different nature of accompanying diseases among older and younger populations, which could have affected the mortality rate. Similar to our findings, Bozorgmanesh et al. in the context of the Tehran Lipid and Glucose Study observed an inverse association between LAP and all‐cause mortality; however, the authors failed to address any association among the female population.^7^ This similarity in findings from two Iranian cohort studies and the variance from findings retrieved from other regions highlights the role of ethnicity in this subject.

Furthermore, we did not find any significant difference between LAP and VAI for CVD incidence, CVD mortality, or all‐cause mortality. Analysis comparing the predictive performance of VAI and LAP with BMI indicated greater AUC for VAI and LAP according to the incidence of CVD; however, considering all‐cause mortality, BMI had significantly greater AUC.

To the best of our knowledge, very limited evidence is available that provides data regarding the discriminatory accuracy of these two indices. Moreover, none of the studies specifically focus on CVDs. Ahn et al. reported a lower discriminatory ability of VAI compared with LAP for diagnosing pre‐diabetes/diabetes.^16^ Similar results have been concluded in MetS^15^ and HTN.^13^, ^14^ The optimal cut‐off values for VAI and LAP in the aforementioned studies differed from ours, which could be due to the difference in the variables and the population characteristics.

Contrary to the majority of the mentioned studies, the present study was conducted in a prospective manner minimizing information and selection bias. Furthermore, the relatively large sample size and the long‐term continuous surveillance of patients' status were the points of strength in this study. Despite the insights that this study might have provided, it is not free from limitations. First, the data are restricted to the people of Persian ancestry living in the central part of Iran; therefore, results might not be capable of being generalized to other ethnicities, and studies on more diverse samples are required to establish the role of VAI and LAP further. Second, due to the self‐reporting nature of questionnaires, recall bias and report bias were possible in the information‐gathering process.

## 5 CONCLUSION

In summary, we observed a significant association between LAP and VAI with CVD incidence, which remained in VAI after adjusting for confounding variables. LAP and VAI were also significantly correlated with SBP, DBP, FBS, HDL‐C, and LDL‐C. In addition, both indices correlated with all‐cause mortality but failed to show a significant correlation with CVD‐specific mortality. According to the ROC curve analyses, neither VAI nor LAP was of poor value for the prognostication of CVD incidence, CVD‐specific mortality, or all‐cause mortality. There was no difference in the discriminatory accuracy of these indices for the observed outcomes. Compared to BMI, VAI and LAP showed greater predictive performance for CVD incidence, but BMI indicated the greatest performance for predicting all‐cause mortality. Nevertheless, more studies with larger sample sizes and more extended follow‐up periods are warranted to reveal whether VAI and LAP are worthy enough to be used in clinical practice.

## CONFLICT OF INTEREST STATEMENT

All authors declare no conflicts of interest.

## Supporting information

Table S1
