# Supplementary material for: Lipid accumulation product and visceral adiposity index for incidence of cardiovascular diseases and mortality; results from 13 years follow‐up in Isfahan cohort study
Source: Obes Sci Pract. 2023 Oct 2;10(1):e713. doi: 10.1002/osp4.713 (PMC10804326; doi:10.1002/osp4.713)
Supplement: Supplementary file 1 — Table S1 [file OSP4-10-e713-s001.docx]

## Supplementary Table 1. Pairwise comparison of ROC curves for CVD incidence

| LAP ~ VAI.final | |
| --- | --- |
| Difference between areas | 0/00201 |
| Standard Error ^a^ | 0/00885 |
| 95% Confidence Interval | -0/0153 to 0/0194 |
| Significance level | P = 0/8207 |
| LAP ~ BMI | |
| Difference between areas | 0/0478 |
| Standard Error ^a^ | 0/0141 |
| 95% Confidence Interval | 0/0202 to 0/0755 |
| Significance level | P = 0/0007 |
| VAI.final ~ BMI | |
| Difference between areas | 0/0458 |
| Standard Error ^a^ | 0/0179 |
| 95% Confidence Interval | 0/0107 to 0/0810 |
| Significance level | P = 0/0106 |

^a^ Hanley & McNeil, 1983

## Supplementary Table 2. Pairwise comparison of ROC curves for all-cause mortality

| LAP ~ VAI.final | |
| --- | --- |
| Difference between areas | 0/00615 |
| Standard Error ^a^ | 0/00901 |
| 95% Confidence Interval | -0/0115 to 0/0238 |
| Significance level | P = 0/4946 |
| LAP ~ BMI | |
| Difference between areas | 0/0430 |
| Standard Error ^a^ | 0/0137 |
| 95% Confidence Interval | 0/0162 to 0/0699 |
| Significance level | P = 0/0017 |
| VAI.final ~ BMI | |
| Difference between areas | 0/0492 |
| Standard Error ^a^ | 0/0180 |
| 95% Confidence Interval | 0/0140 to 0/0844 |
| Significance level | P = 0/0062 |

## Supplementary Table 3. Pairwise comparison of ROC curves for CVD associated mortality

| LAP ~ VAI.final | |
| --- | --- |
| Difference between areas | 0/00224 |
| Standard Error ^a^ | 0/0140 |
| 95% Confidence Interval | -0/0252 to 0/0297 |
| Significance level | P = 0/8730 |
| LAP ~ BMI | |
| Difference between areas | 0/0321 |
| Standard Error ^a^ | 0/0203 |
| 95% Confidence Interval | -0/00778 to 0/0719 |
| Significance level | P = 0/1147 |
| VAI.final ~ BMI | |
| Difference between areas | 0/0298 |
| Standard Error ^a^ | 0/0268 |
| 95% Confidence Interval | -0/0227 to 0/0824 |
| Significance level | P = 0/2658 |

^a^ Hanley & McNeil, 1983
